# Supplementary material for: Manipulating the diffusion energy barrier at the lithium metal electrolyte interface for dendrite-free long-life batteries
Source: Nat Commun. 2024 Apr 10;15:3085. doi: 10.1038/s41467-024-47521-z (PMC11006908; doi:10.1038/s41467-024-47521-z)
Supplement: Supplementary file 1 — Supplementary Information [file 41467_2024_47521_MOESM1_ESM.pdf]

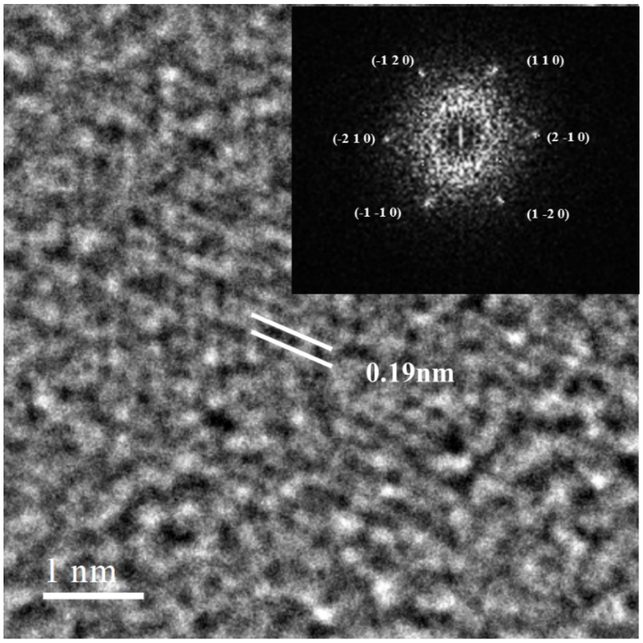

Supplementary Fig. 1. Transmission electron microscopy (TEM) image and corresponding diffraction patterns of TEMED treated lithium.

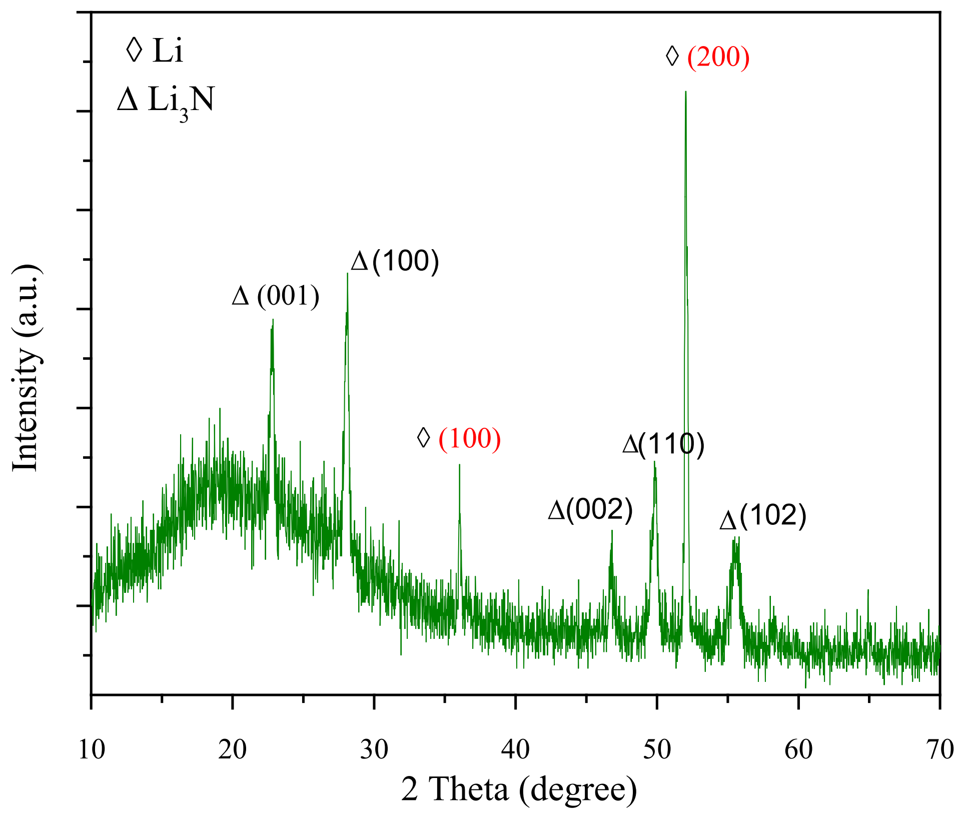

Supplementary Fig. 2. XRD pattern of Li_3N film obtained under N_2 environment.

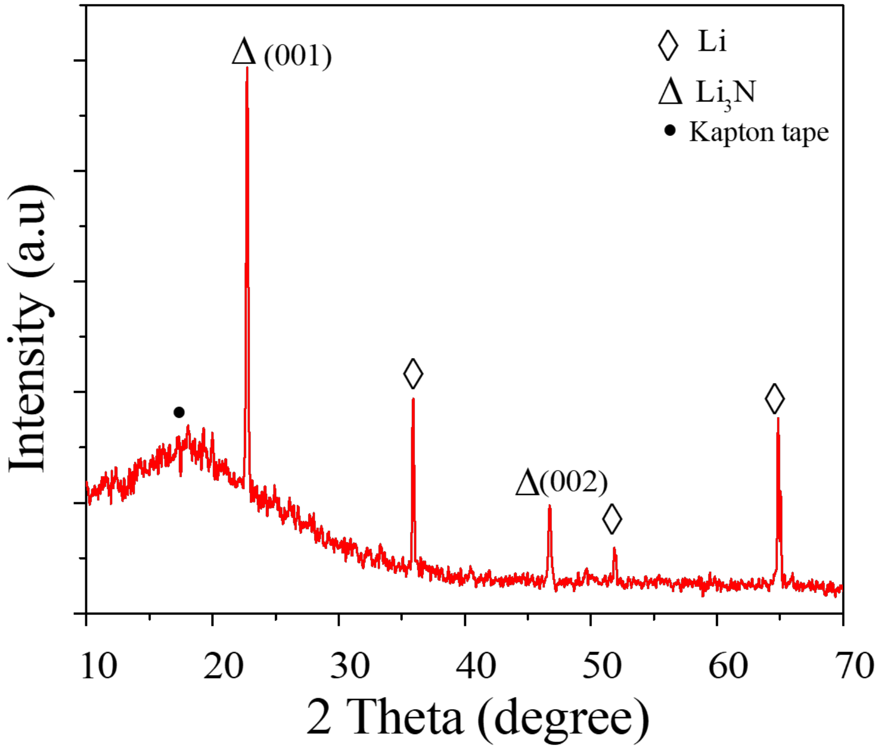

Supplementary Fig. 3. XRD pattern of TEMED-treated Li^0 after 100 cycles at a current of 0.5 mA cm^{-2} and with a total capacity of 1 mAh cm^{-2}.

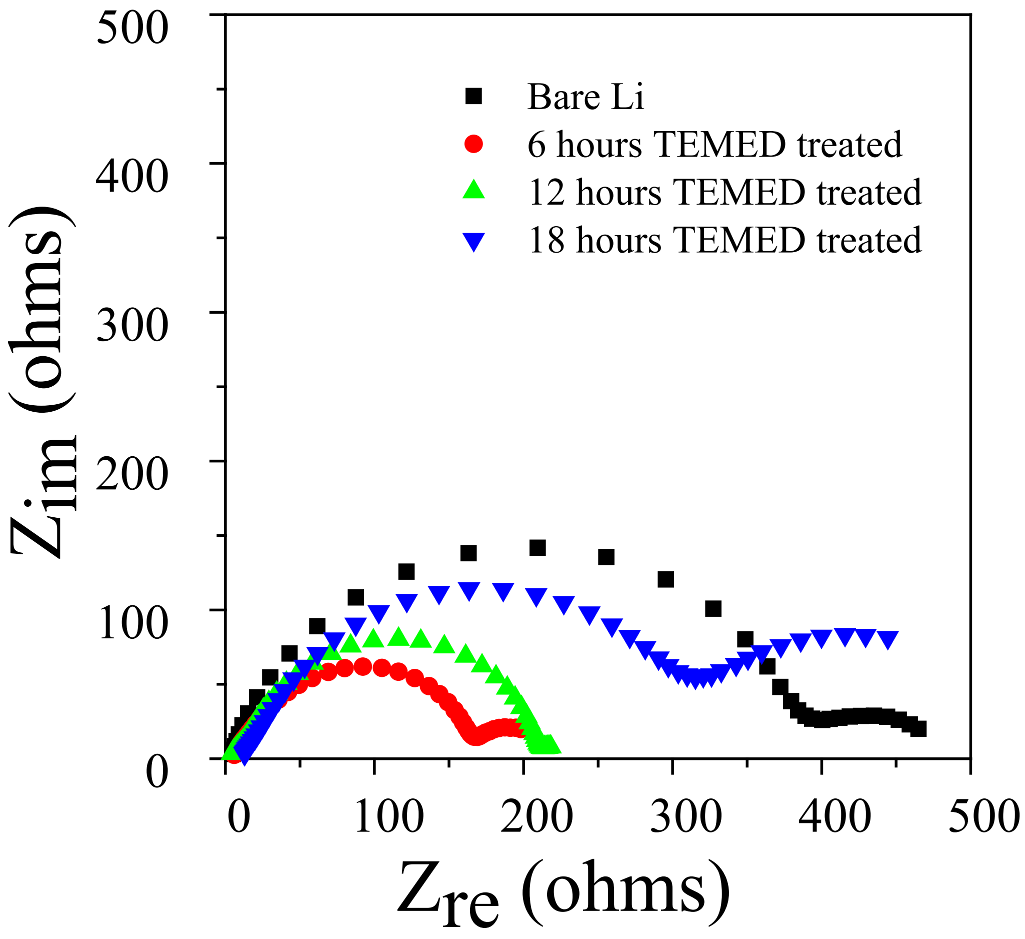

Supplementary Fig. 4. Comparative EIS measurement for different treatment time for TEMED treated Li^0 and untreated Li^0.

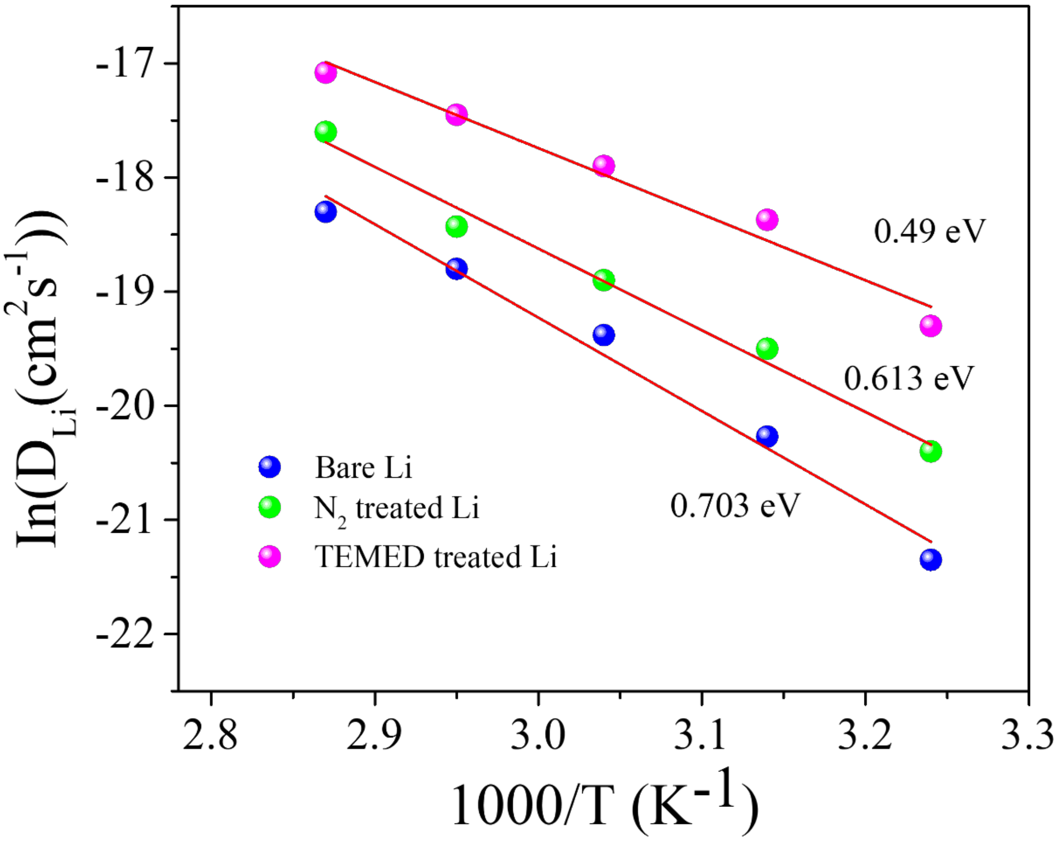

Supplementary Fig. 5. Correlation between ln D and reciprocal temperature (Arrhenius-plot) for TEMED treated Li^0 and untreated Li^0. The ionic conductivity of Li_3N can be calculated using the equation $\sigma = 2\,L/Ra$, in which, L is the thickness of Li_3N, R is the resistance of Li_3N and a is the area.

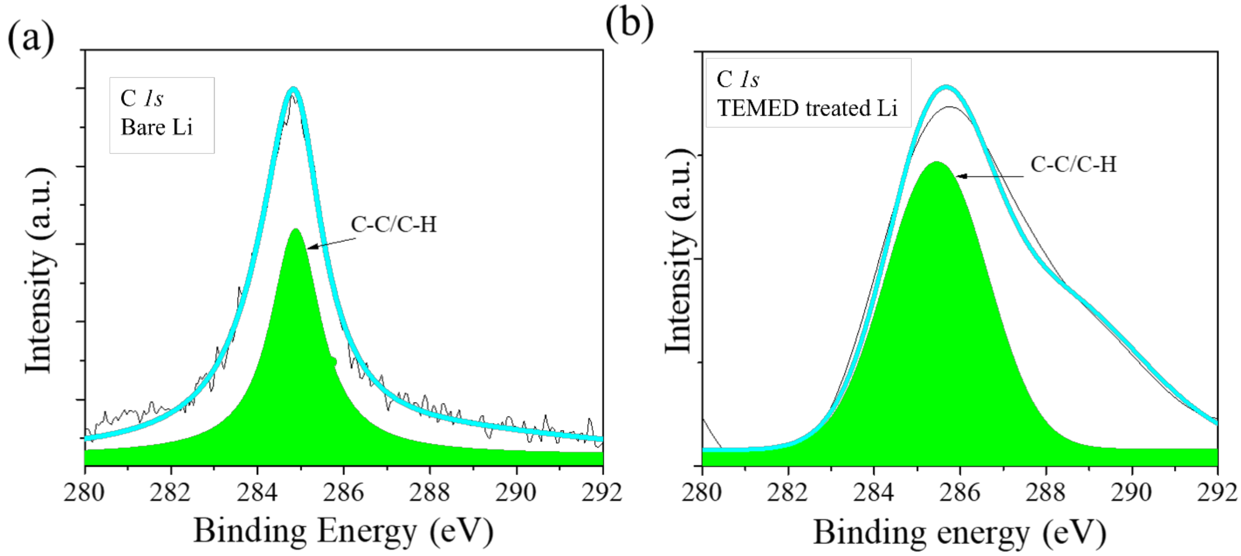

Supplementary Fig. 6. (a) XPS C *1s* spectra for Bare Li^0. (**b)** C *1s* spectra for TEMED-treated Li^0.

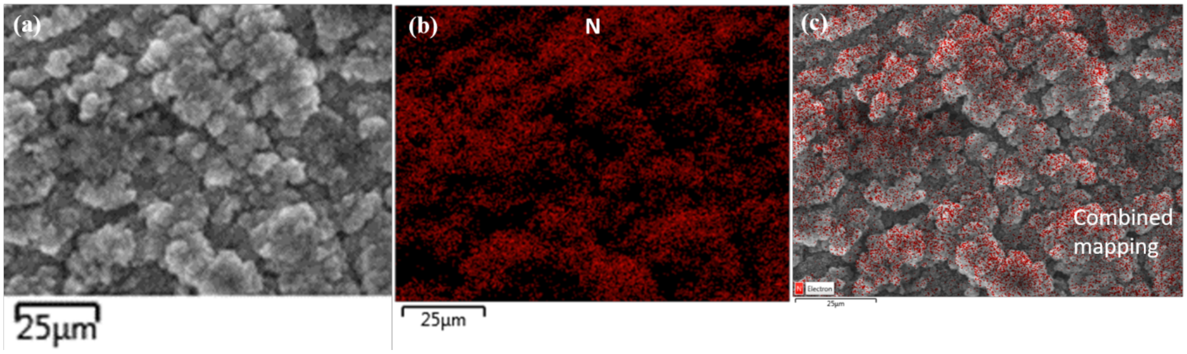

Supplementary Fig. 7. **(a)** Scanning electron microscope (SEM) image of the surface of TEMED-treated Li^0. **(b)** The corresponding energy dispersive spectroscopy (EDS) mapping (nitrogen) in the same area. (c) The combined SEM image and the EDS mapping showing a uniform distribution of nitrogen over the surface of the TEMED-treated Li^0.

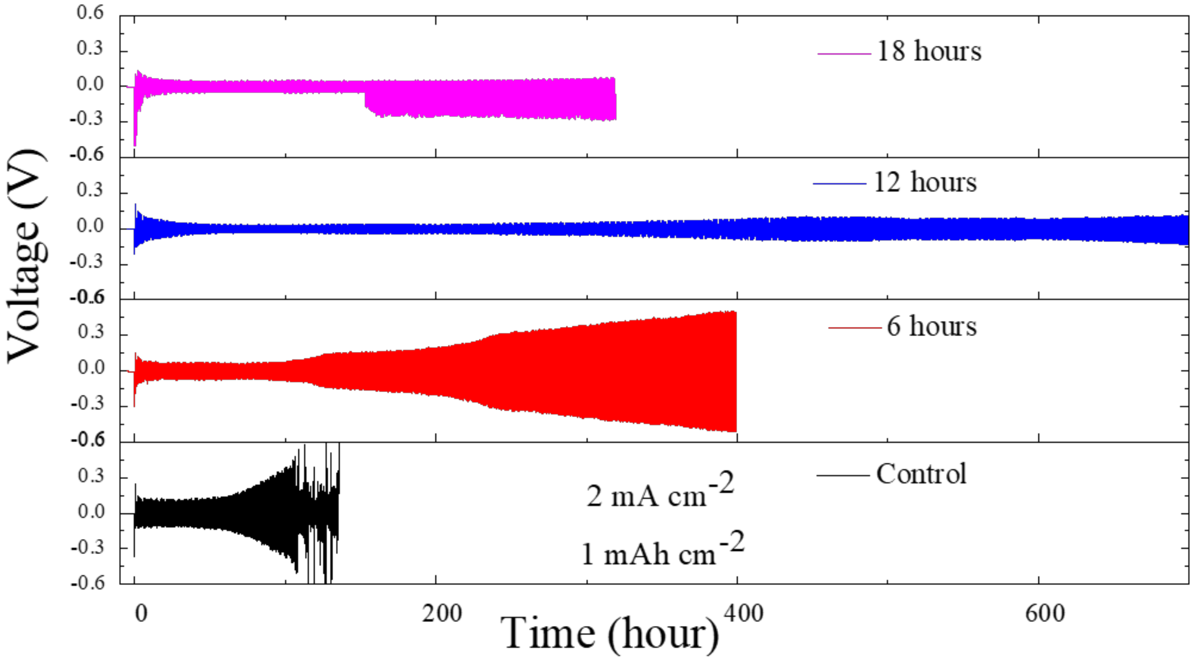

Supplementary Fig. 8. The voltage profiles of symmetrical cells with untreated Li^0 and TEMED-treated Li^0 with different treatment time at a current density of 2 mA cm^{-2} and a capacity of 1 mAh cm^{-2}.

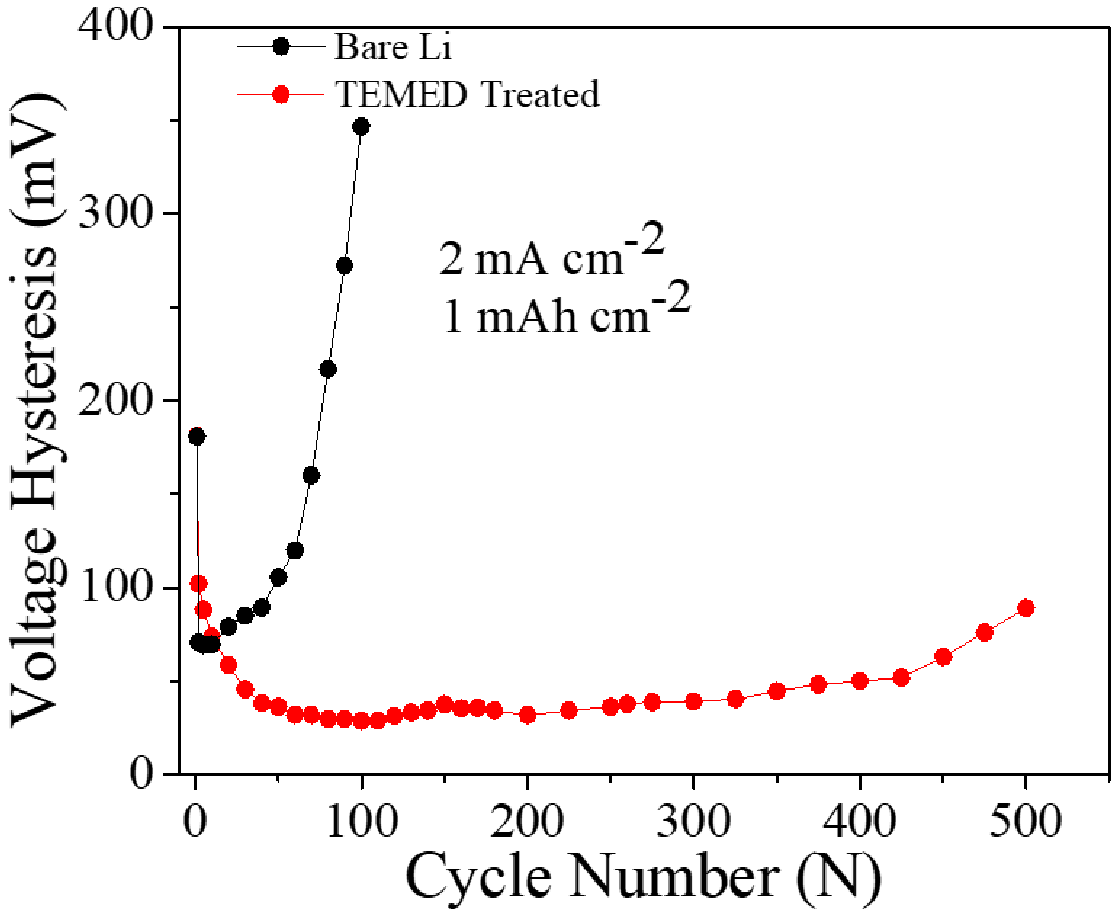

Supplementary Fig. 9. Comparative voltage profiles of symmetrical cells based on 12-hour TEMED treated Li^0 and untreated Li^0 at a current density of 2 mA cm^{-2} and a capacity of 1 mAh cm^{-2}.

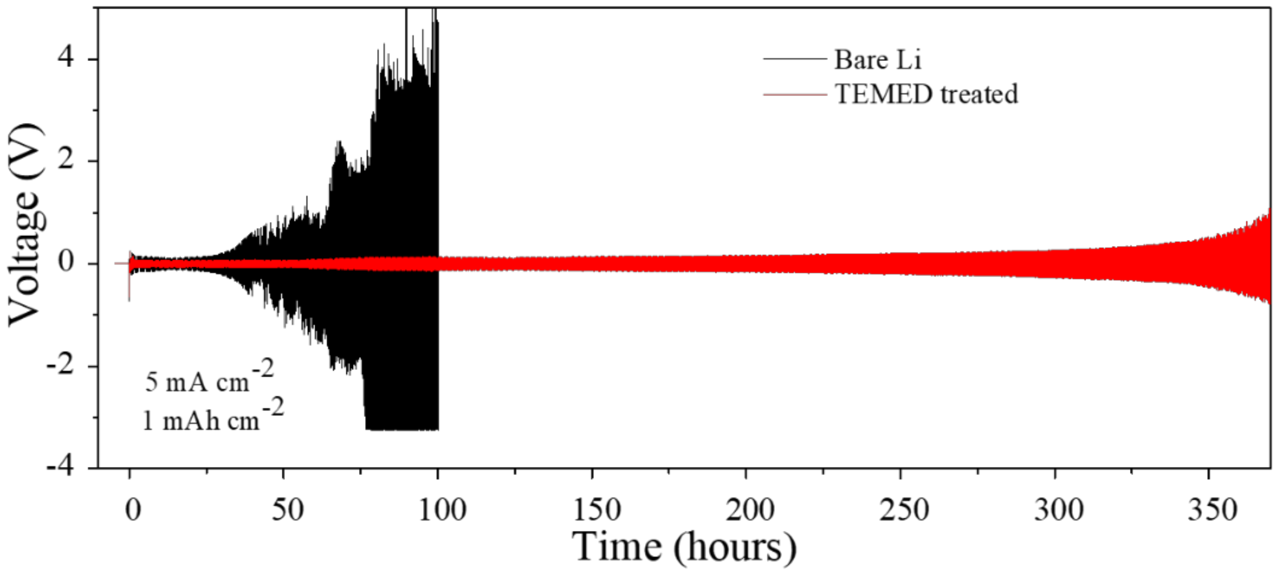

Supplementary Fig. 10. The voltage profiles of symmetrical cells based on 12-hour TEMED treated Li^0 and untreated Li^0 at a current density of 5 mA cm^{-2} and a capacity of 1 mAh cm^{-2}.

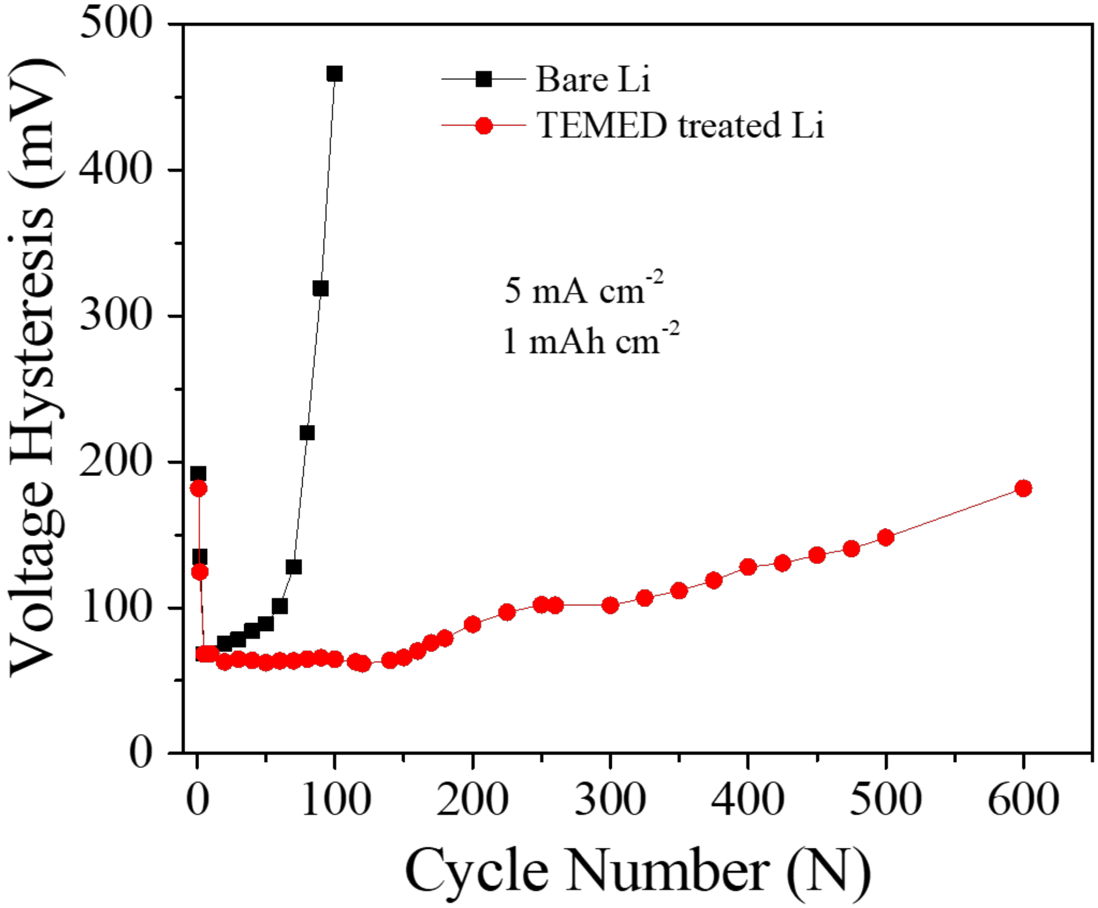

Supplementary Fig. 11. Comparative voltage profiles of TEMED treated symmetrical cells based on 12-hour TEMED treated Li^0 and untreated Li^0 at a current density of 5 mA cm^{-2} and a capacity of 1 mAh cm^{-2}.

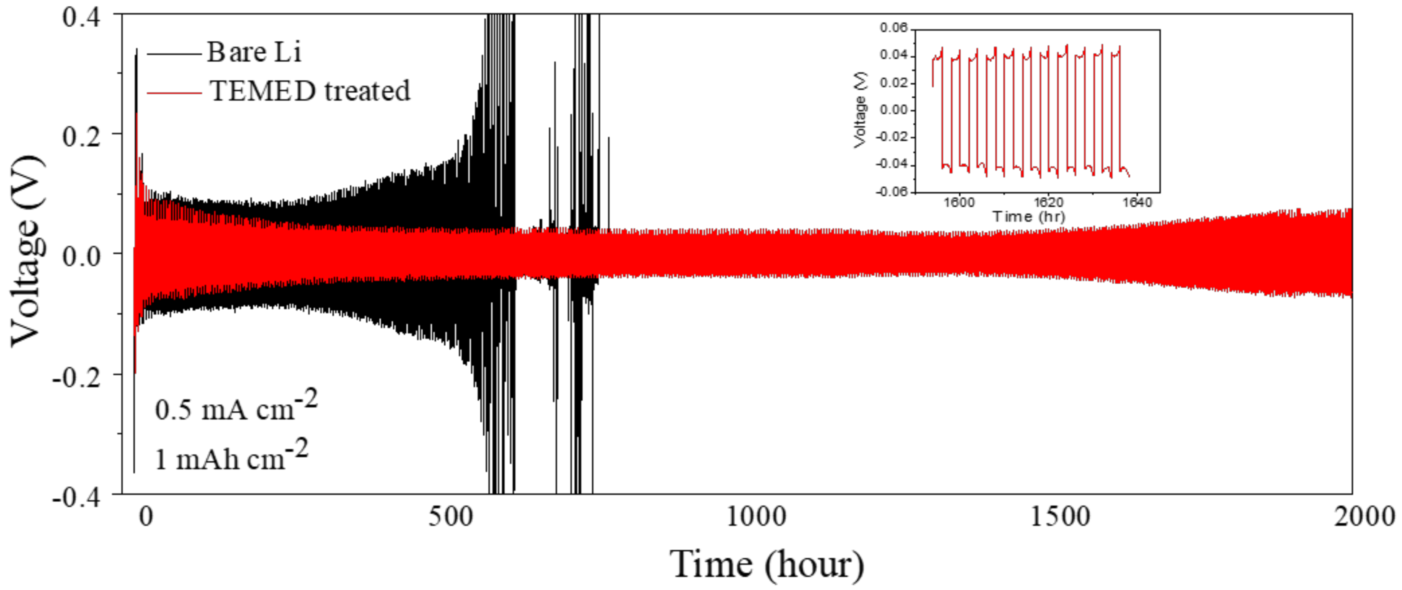

Supplementary Fig. 12. The voltage profiles of symmetrical cells based on TEMED-treated Li^0 and untreated Li^0 at a current density of 0.5 mA cm^{-2} and a capacity of 1 mAh cm^{-2} using 1M $LiPF_6$ in EC/DEC as an electrolyte.

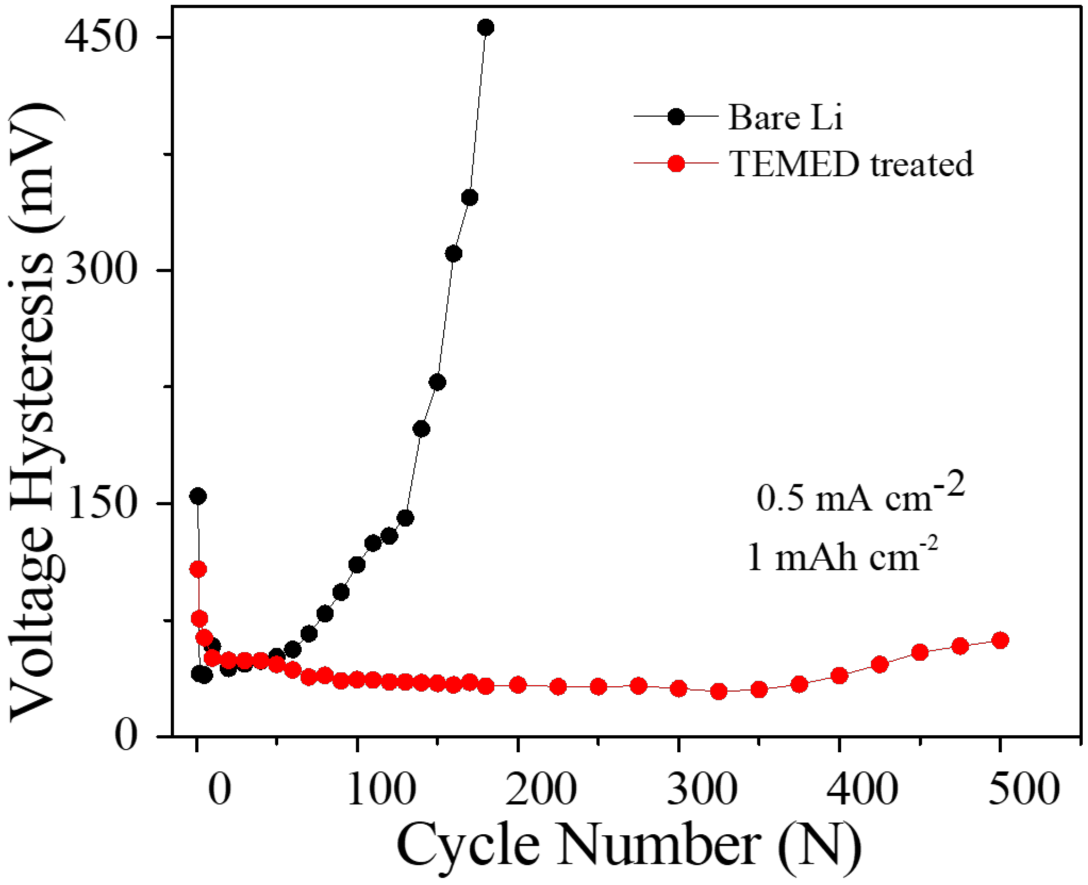

Supplementary Fig. 13. Comparative voltage profiles of symmetrical cells based on TEMED-treated Li^0 and untreated Li^0 at a current density of 0.5 mA cm^{-2} and a capacity of 1 mAh cm^{-2} using 1M $LiPF_6$ in EC/DEC as an electrolyte..

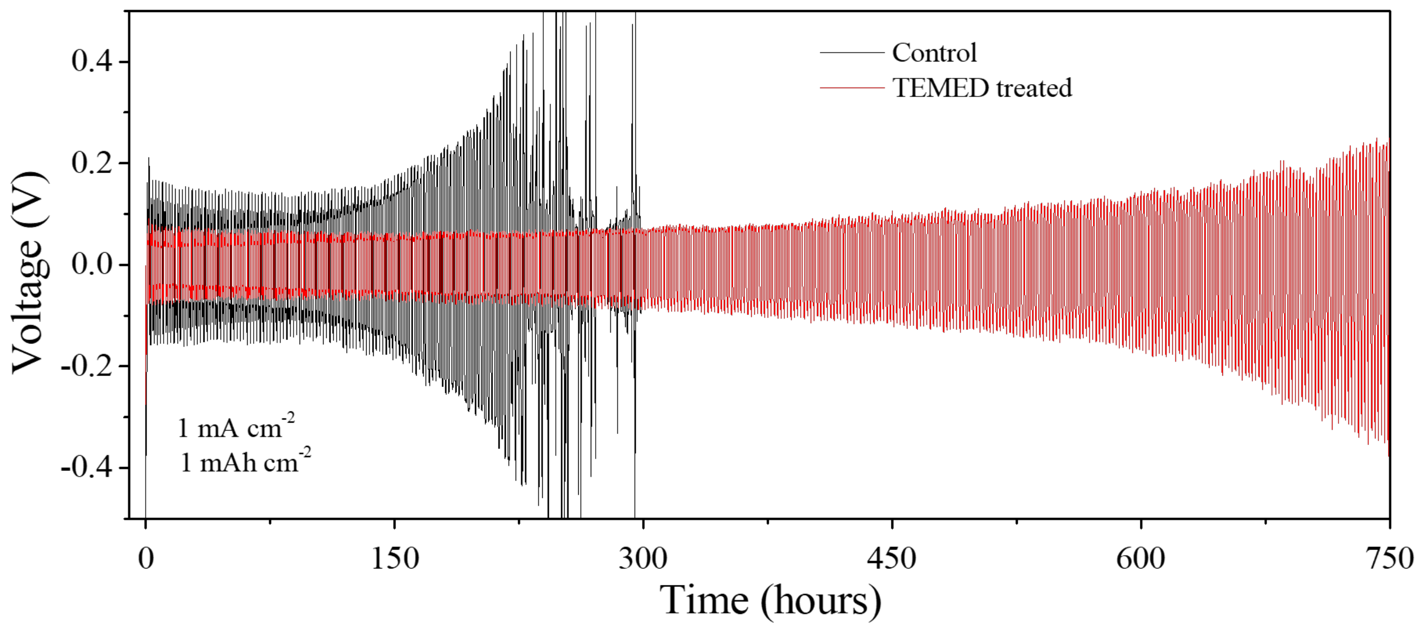

Supplementary Fig. 14. The voltage profiles of symmetrical cells based on TEMED-treated Li^0 and untreated Li^0 at a current density of 1 mA cm^{-2} and a capacity of 1 mAh cm^{-2} using 1M $LiPF_6$ in EC/DEC as an electrolyte.

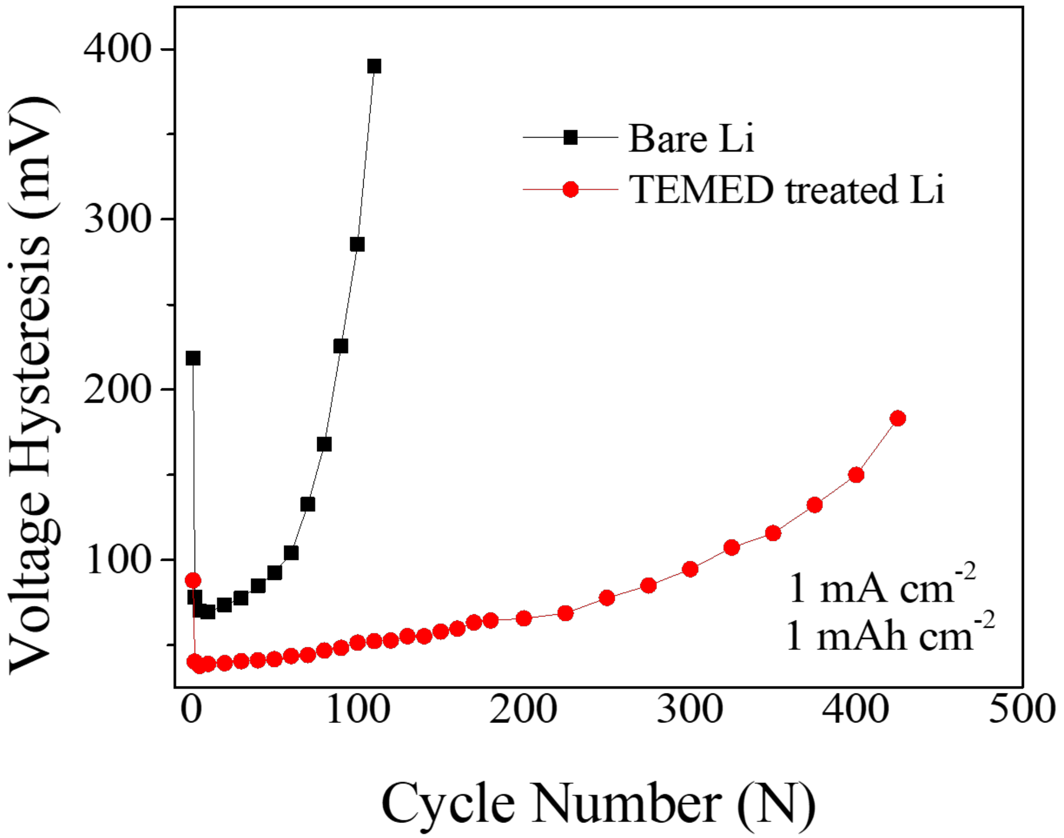

Supplementary Fig. 15. Comparative voltage profiles of symmetrical cells based on TEMED-treated Li^0 and untreated Li^0 at a current density of 1 mA cm^{-2} and a capacity of 1 mAh cm^{-2} using 1M $LiPF_6$ in EC/DEC as an electrolyte..

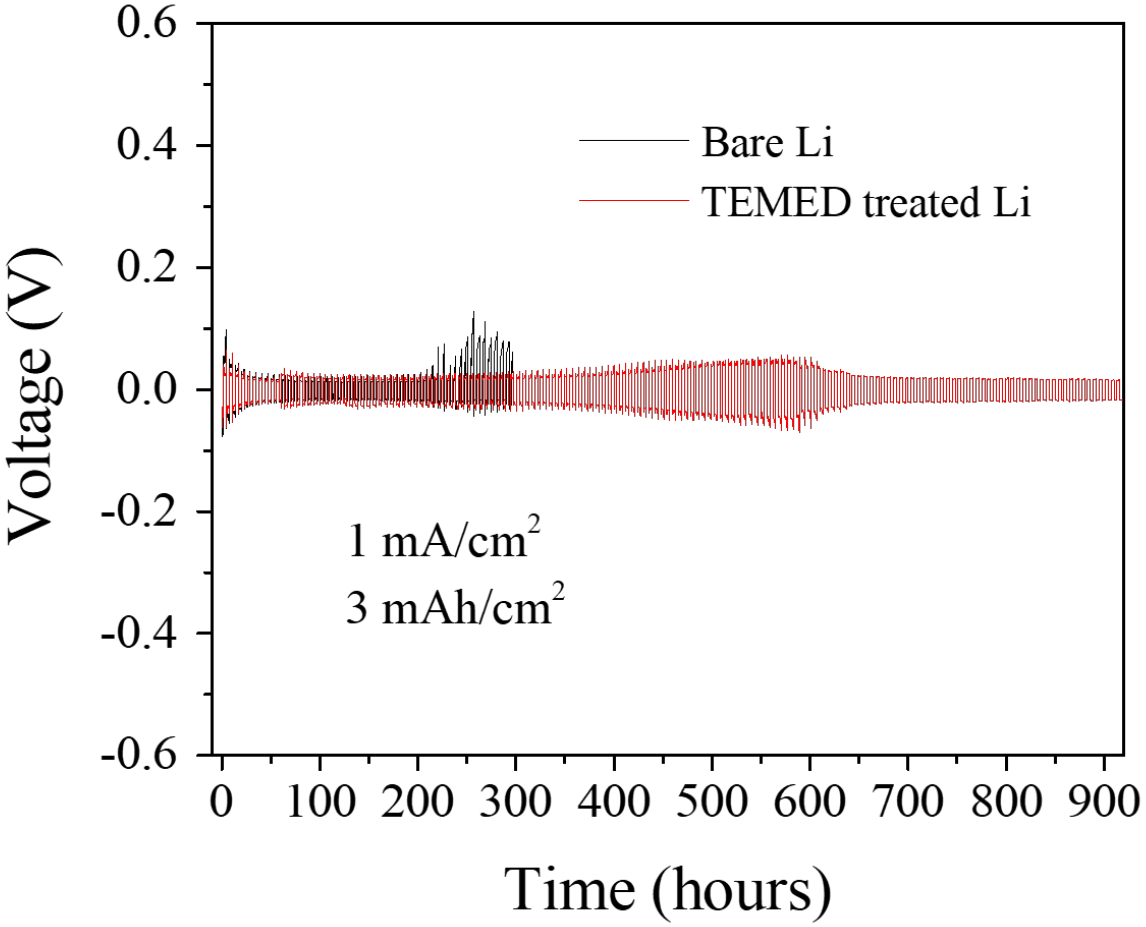

Supplementary Fig. 16. Comparative voltage profiles of symmetrical cells based on TEMED-treated Li^0 and untreated Li^0 at a current density of 1 mA cm^{-2} and a capacity of 3 mAh cm^{-2} with 50 um Li chip.

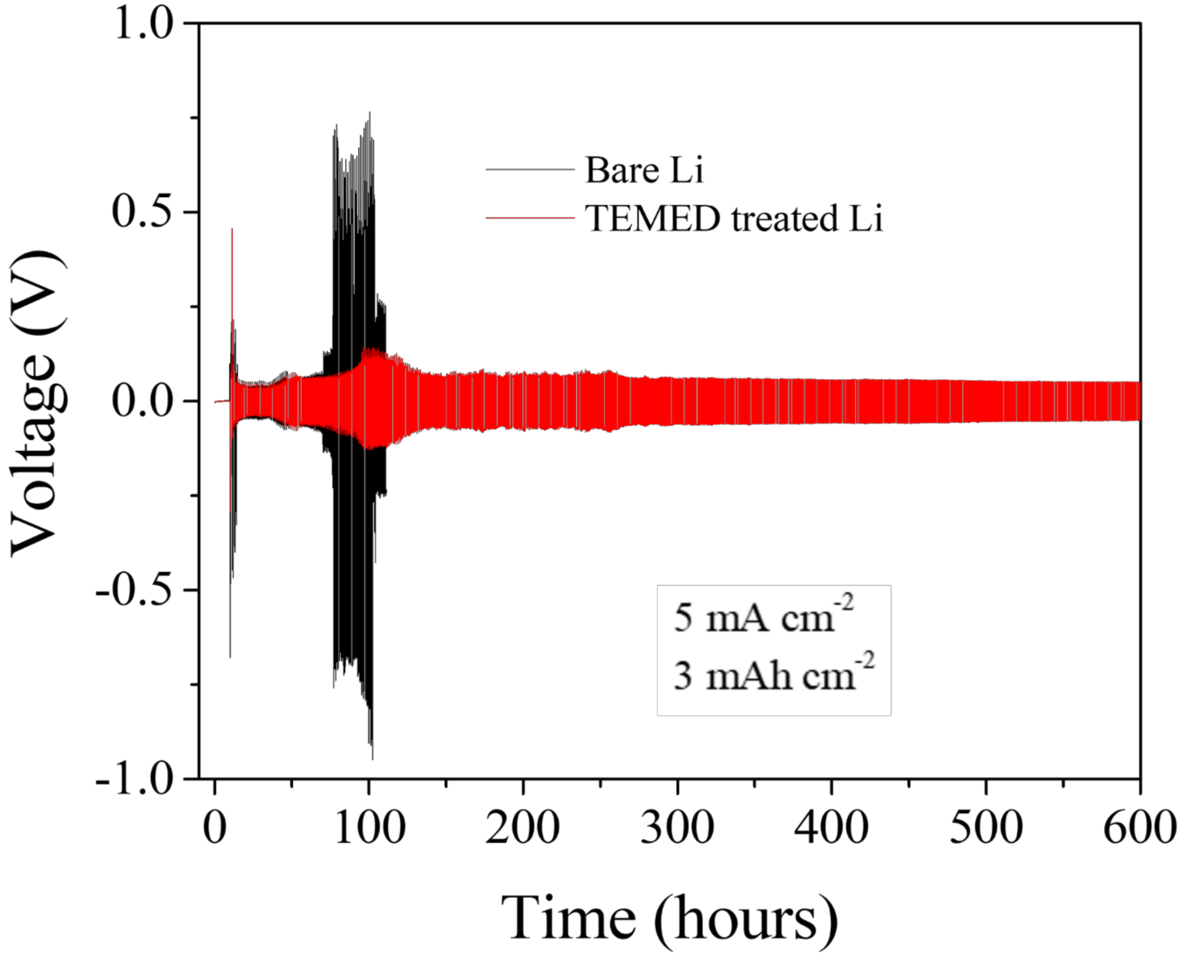

Supplementary Fig. 17. Comparative voltage profiles of symmetrical cells based on TEMED-treated Li^0 and untreated Li^0 at a current density of 5 mA cm^{-2} and a capacity of 3 mAh cm^{-2} with 50 um Li chip.

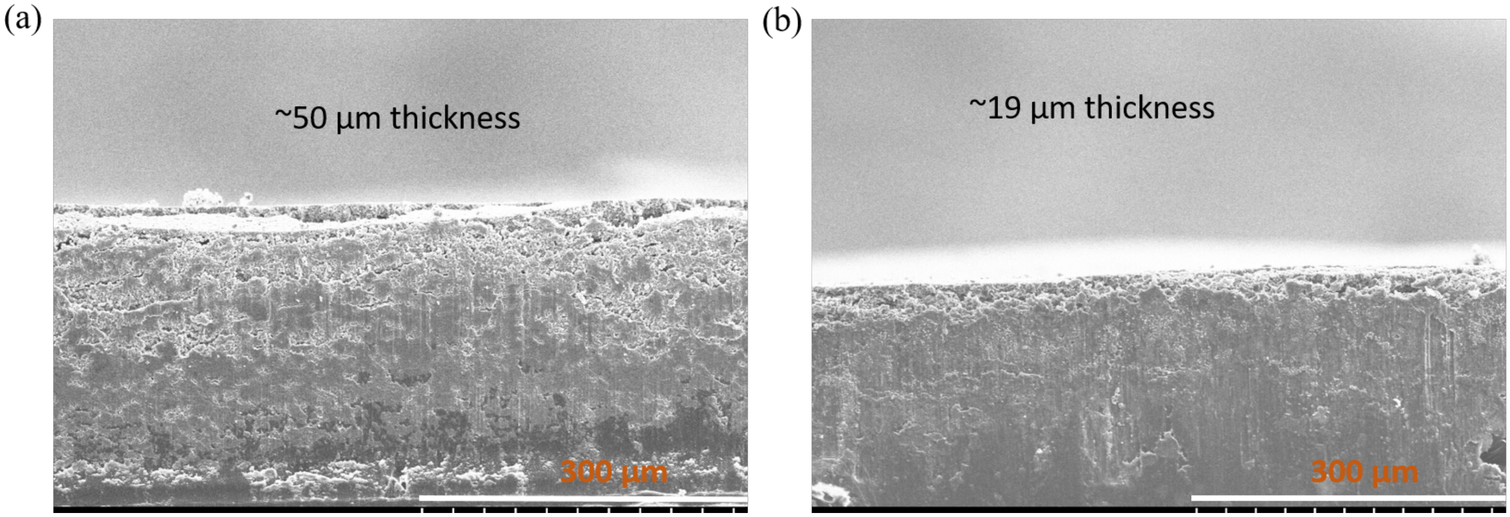

Supplementary Fig. 18. Cross sectional SEM images of **(a)** untreated Li^0 and **(b)** TEMED-treated Li^0 after 100 cycles of charge/discharge.

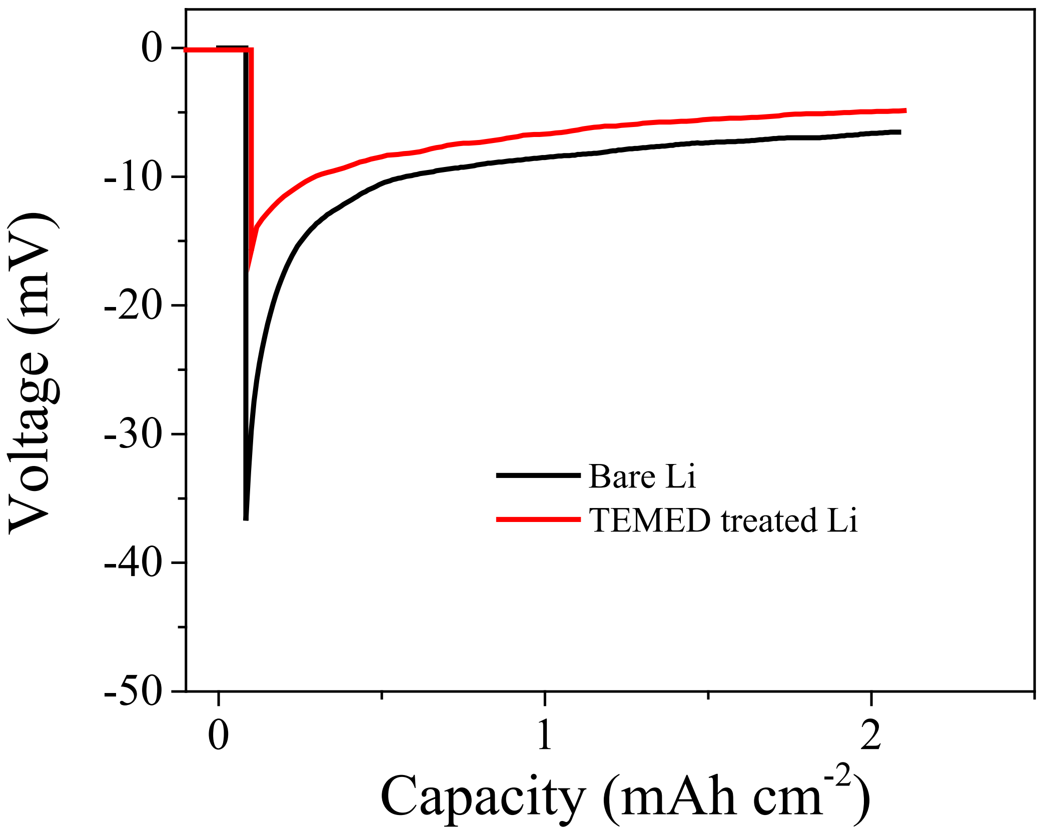

Supplementary Fig. 19. Nucleation overpotential comparison of untreated Li^0 and TEMED treated Li^0.

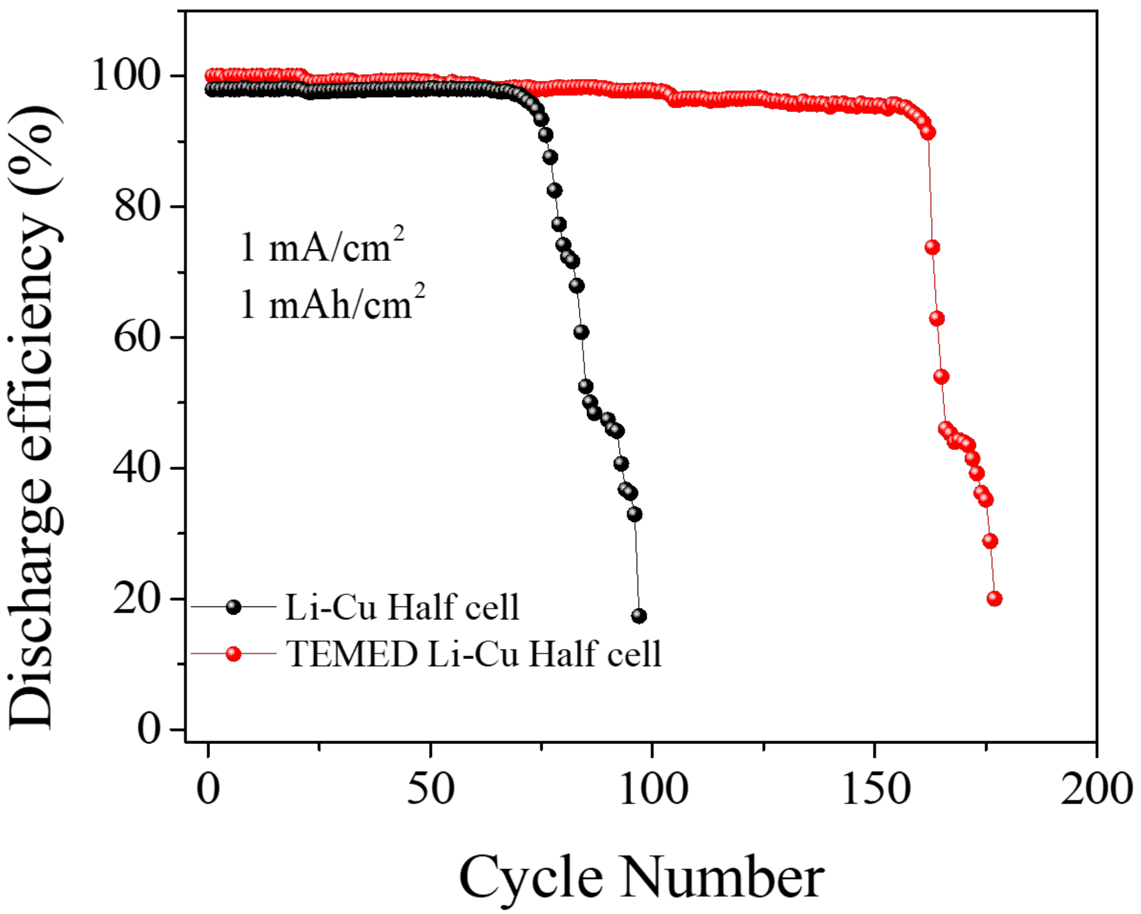

Supplementary Fig. 20. Half-cell performance of the Li-Cu and TEMED Li-Cu cells at a current density of 1 mA cm^{-2} with a capacity of 1 mAh cm^{-2}. The thickness of Li chip is 50 μm.

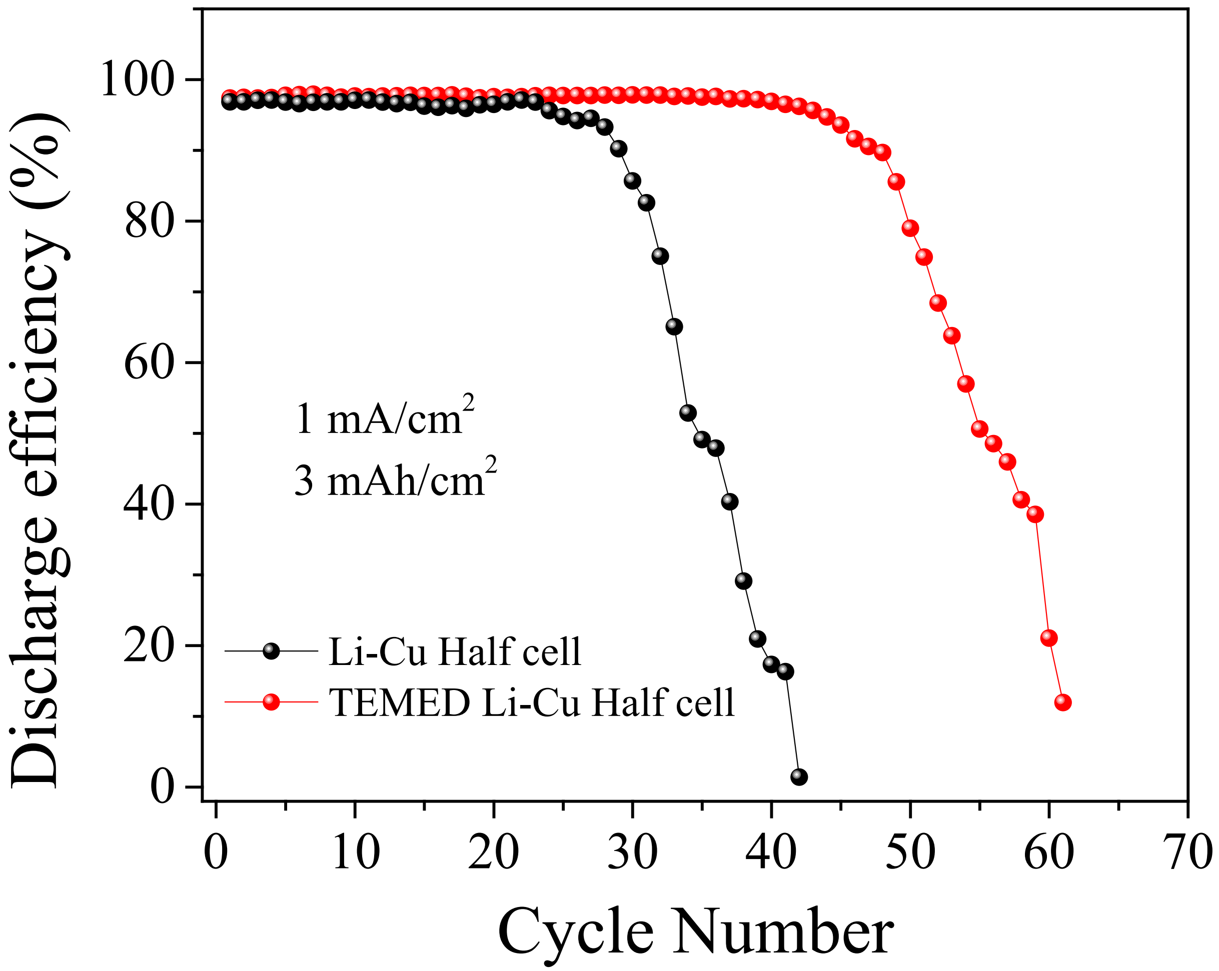

Supplementary Fig. 21. Half-cell performance of the Li-Cu and TEMED Li-Cu at a current density of 1 mA cm^{-2} with a capacity of 3 mAh cm^{-2}. The thickness of Li chip is 50 μm.

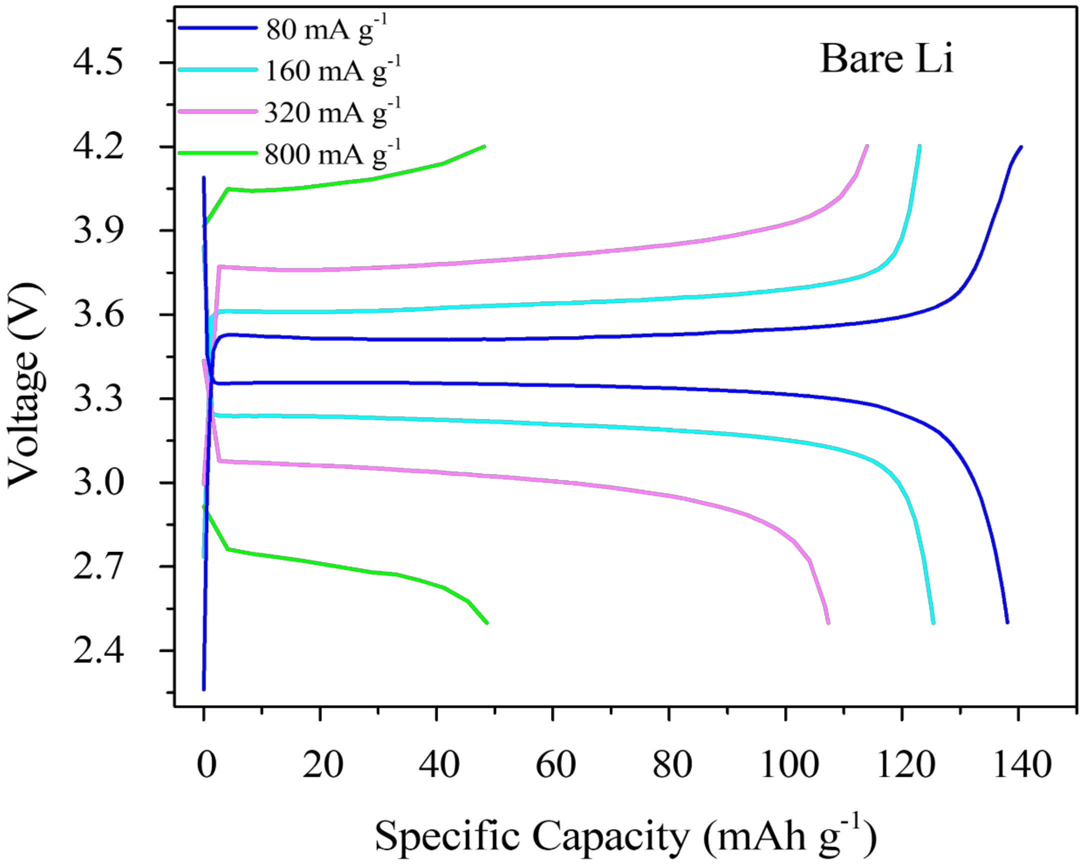

Supplementary Fig. 22. Charge/discharge voltage profiles of full cells using LFP coupled with untreated Li^0 as anode at different specific currents. The mass loading of LFP is ~2 mg cm^{-2}.

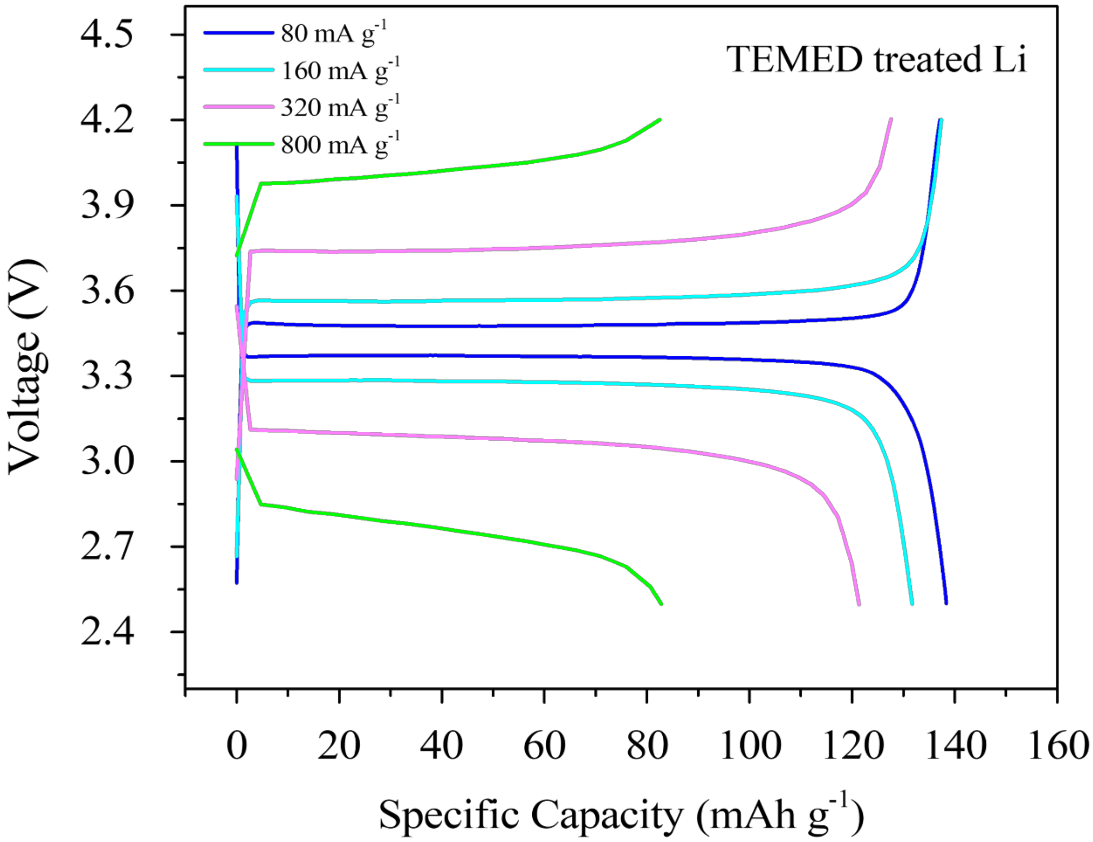

Supplementary Fig. 23. Charge/discharge voltage profiles of full cells using LFP coupled with TEMED-treated Li^0 as anode at different current densities. The mass loading of LFP is ~2 mg cm^{-2}.

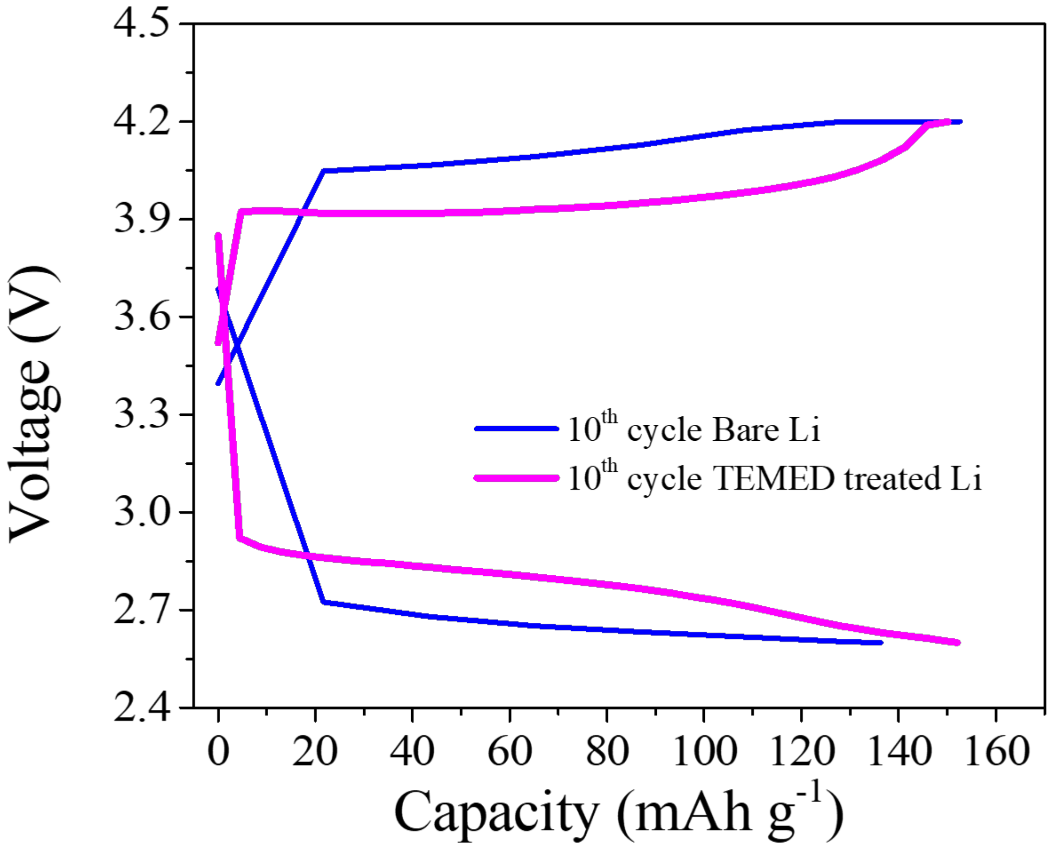

Supplementary Fig. 24. Charge/discharge voltage profiles at 10th cycle of full cells using LFP coupled with untreated Li^0 and TEMED-treated Li^0 at 140 mA g^{-1}. The mass loading of LFP is ~9.5 mg cm^{-2}.

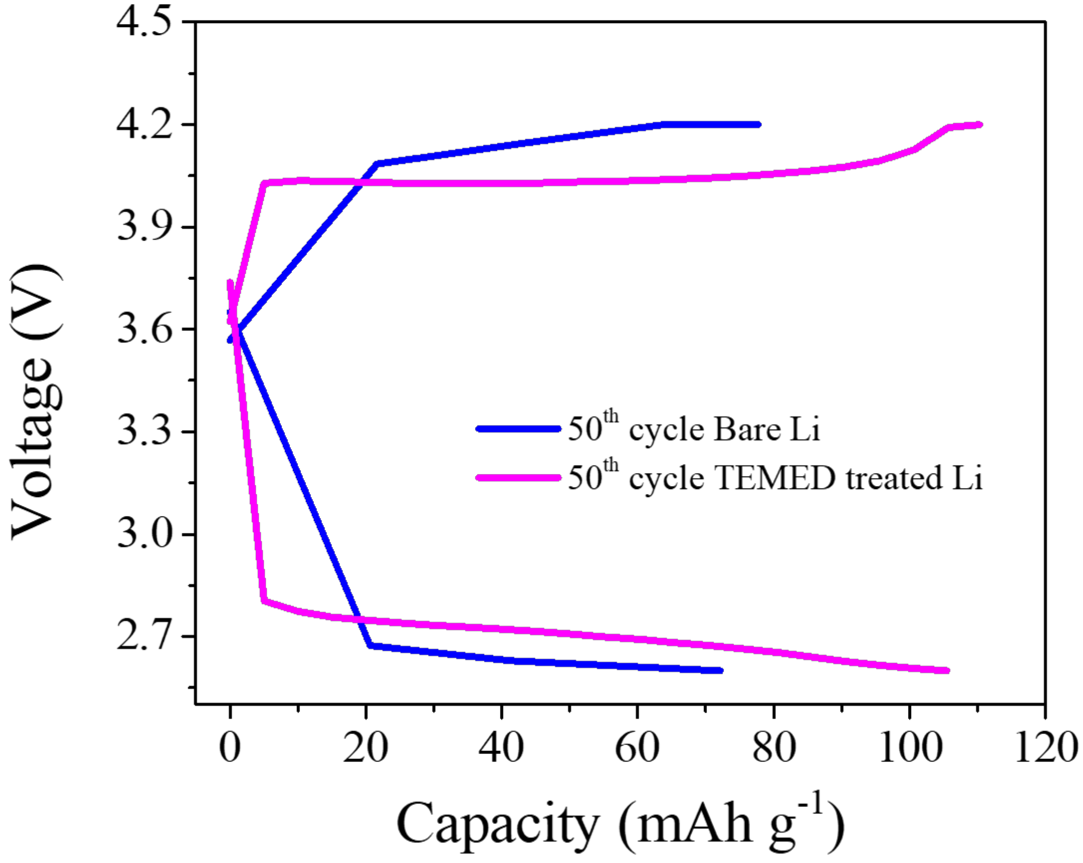

Supplementary Fig. 25. Charge/discharge voltage profiles at 50th cycle of full cells using LFP coupled with untreated Li^0 and TEMED treated Li^0 at 140 mA g^{-1}. The mass loading of LFP is ~9.5 mg cm^{-2}.

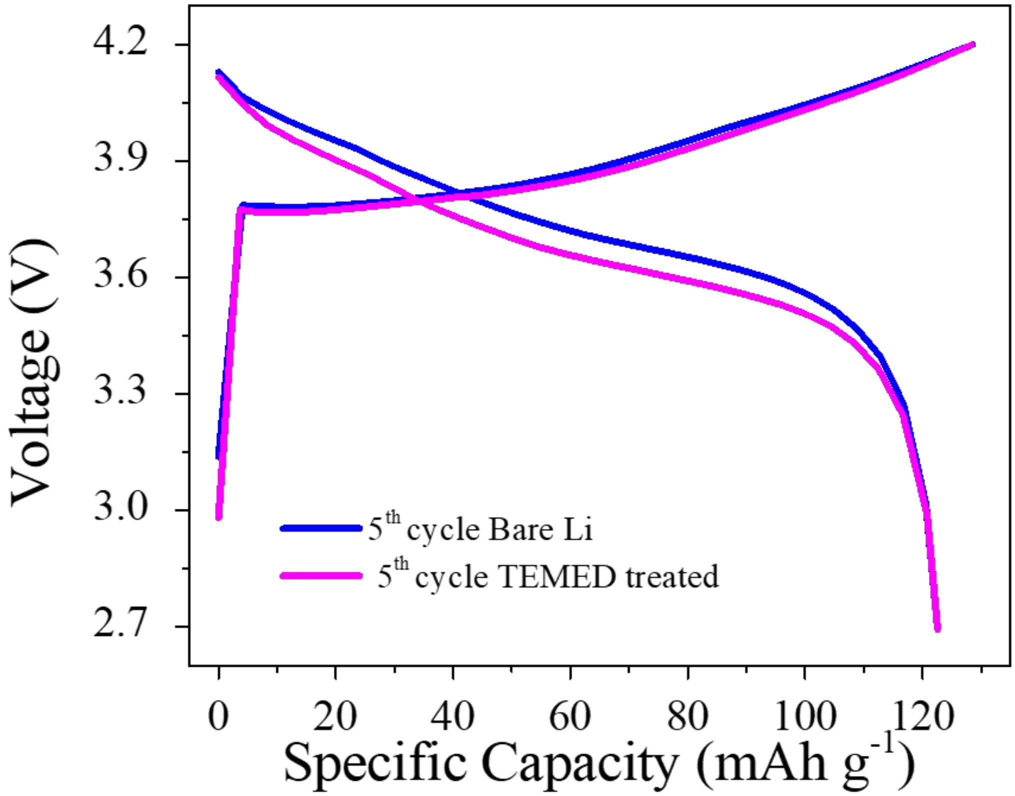

Supplementary Fig. 26. Charge/discharge voltage profiles at 5th cycle of full cells using NMC coupled with untreated Li^0 and TEMED treated Li^0 at 140 mA g^{-1}. The mass loading of NMC is ~2.5 mg cm^{-2}.

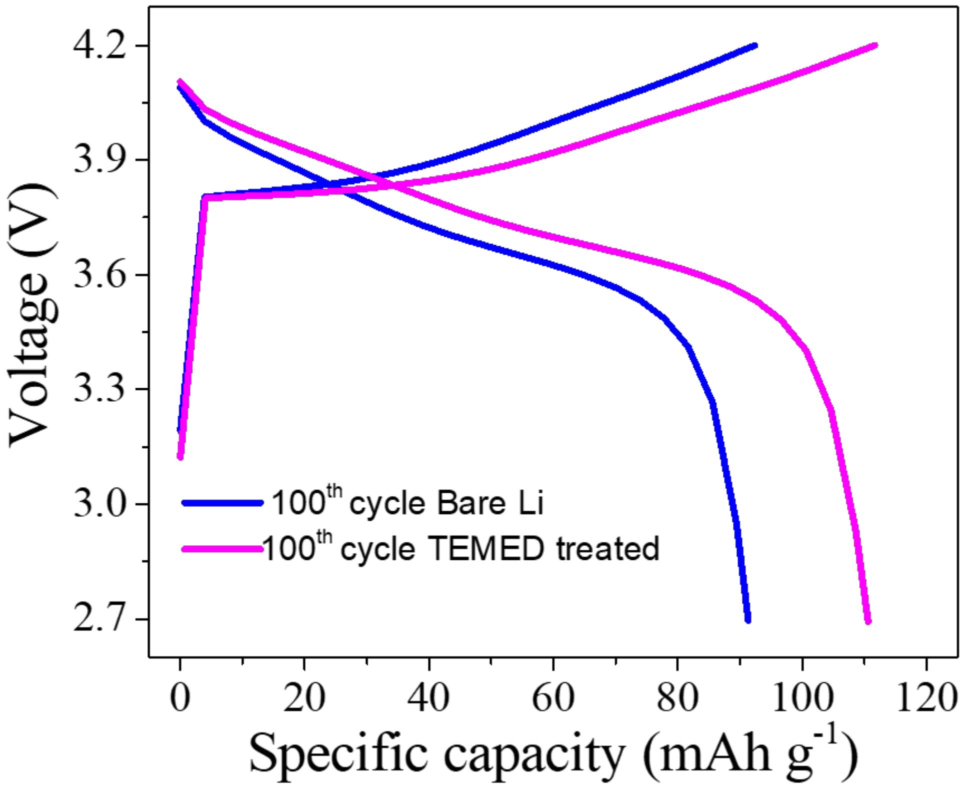

Supplementary Fig. 27. Charge/discharge voltage profiles at 100th cycle of full cells using NMC coupled with untreated Li^0 and TEMED treated Li^0 at 140 mA g^{-1}. The mass loading of NMC is ~2.5 mg cm^{-2}.

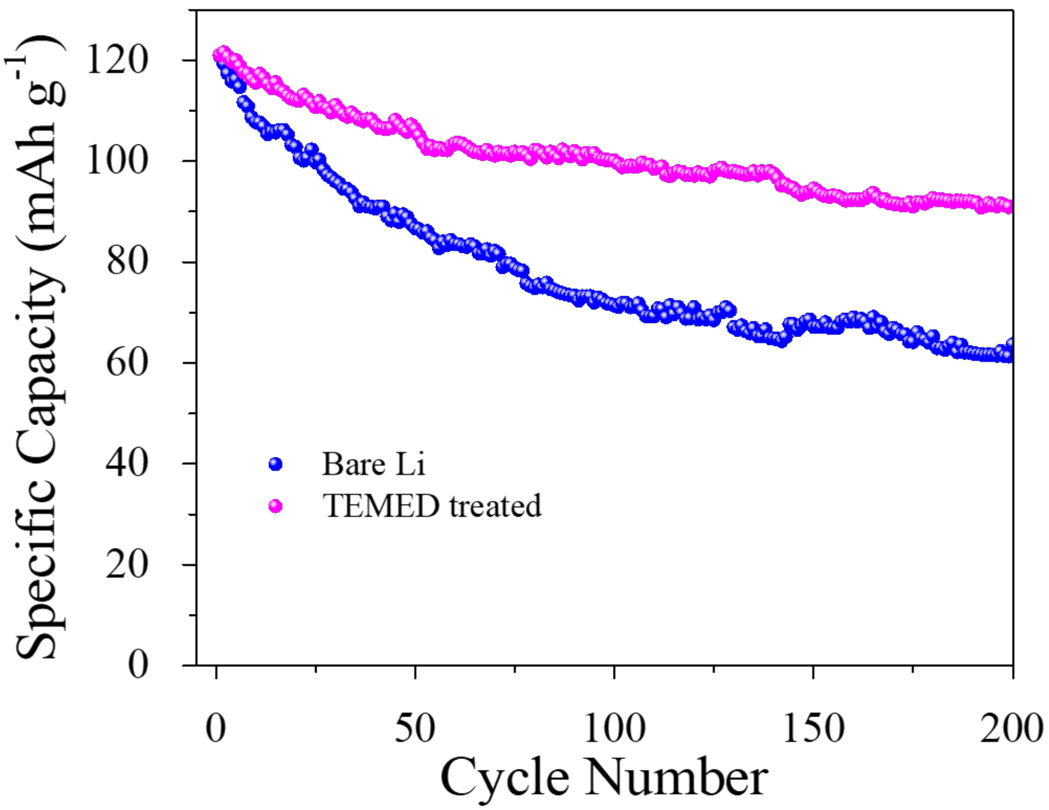

Supplementary Fig. 28. Long-term cycling performance of full cells using NMC coupled with untreated Li^0 and TEMED treated Li^0 at a current density of 140 mA g^{-1}. The mass loading of NMC is ~2.5 mg cm^{-2}.

Lateral and vertical diffusion

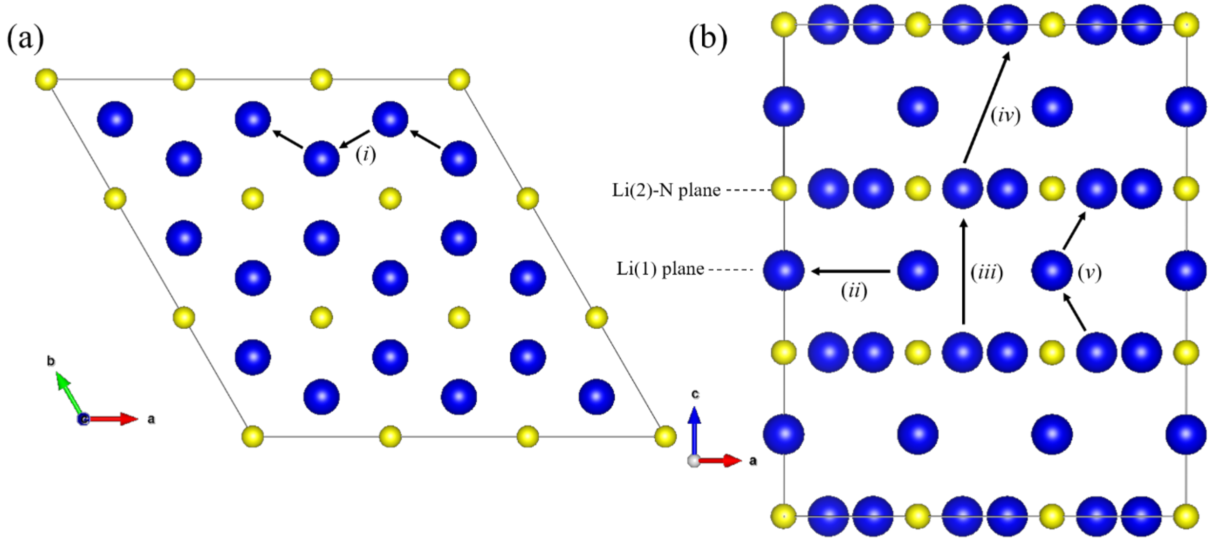

Supplementary Fig. 29. (a) Top view and **(b)** side view of the supercell (3×3×3) structure of a-Li_3N, and possible Li migration pathways.

To investigate the Li diffusion in the α-Li_3N layer, we estimated migration energy barriers by performing density functional theory (DFT) calculations. We considered all possible migration pathways including lateral (or in-plane; ⊥ *c* axis) and vertical (or out-of-plane; || *c* axis) diffusions (Fig. 29). For the lateral diffusion, a Li can diffuse along the Li(2)-N plane (path *i*) or the Li(1) plane (path *ii*), where the diffusion *via* path *i* shows much lower energy barrier (0.01 eV vs. 1.0 eV). For the vertical diffusion, a Li can diffuse between the Li(2)-N plane directly (paths *iii* and *iv*) or passing through the Li(1) plane (path *v*). Paths *iii* and *iv* show ~0.6 eV of energy barrier, whereas path *v* shows 1.8 eV. Because of the lowest barrier, the lateral diffusion *via* path *i* is most likely dominant for Li diffusion in α-Li_3N. Also, the results indicate that the Li diffusion passing through the Li (1) plane has a high barrier for diffusion, and thus Li in the Li(2)-N plane is

responsible for the most diffusion. The diffusion energy along different paths can be found in Supplementary Table1.

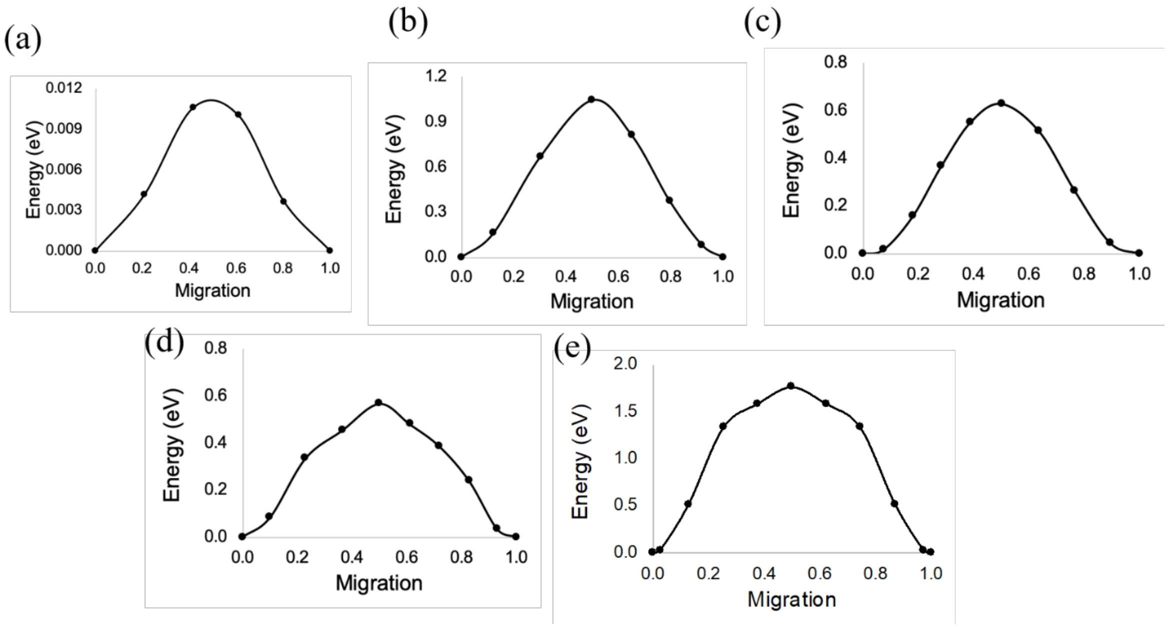

Supplementary Fig. 30. The estimated energy profiles for the Li migration in α -Li_3N; **(a)** path *i*, **(b)** path *ii*, **(c)** path *iii*, **(d)** path *iv*, and **(e)** path *v*, respectively (each pathway is indicated in Fig. x1). Only one step migration was considered for path i consisting of equivalent steps, and the half step migration was calculated (and doubled for plotting) for path v consisting of two equivalent steps.

Supplementary Note 1

Sand's equation

$$t_{Sand} = \pi D_{app} \frac{(Z_c\, c_o\, F)^2}{4(J t_a)^2} \quad (1)$$

Where z_c is the charge number of the cation, c_o is the bulk salt concentration, F is the Faraday's constant, J is current density and $t_a = 1- t_{Li}$.

Supplementary Note 2

Calculation of diffusion coefficient

Li ion diffusion is a key factor which influences the electrochemical performance of the battery. The temperature dependence of Li ion diffusion coefficient (D) was determined by the EIS. The low frequency region of the EIS relates to the Warburg impedance (Z_W) was employed to determine the diffusion coefficient. The Warburg impedance coefficient can be derived from linear fitting of Z-real vs. $\omega^{-1/2}$ (= $(2\pi f)^{-1/2}$)[1]. The diffusion coefficient was then obtained as

$$D_{Li} = \frac{R^2 T^2}{2A^2 F^4 \sigma_\omega^2\, C^2} \quad (2)$$

Where, R is the gas constant (8.314 $J.mol^{-1}.K^{-1}$), T is the absolute temperature (K), A is the surface area of the electrode, F is the Faraday constant (96,485 C/mol), σ_ω is the Warburg impedance coefficient and C is the molar concentration of Li ion in the electrode.

Supplementary Note3

Arrhenius equation

The dependence of diffusion coefficient D on temperature T can be approximately described by an Arrhenius relationship as

$D_{Li} = D_o \, exp\left(-\frac{E_a}{RT}\right)$ (3)

Where, D_{Li} is the diffusion coefficient ($m^2 \cdot s^{-1}$), D_o is the temperature independent pre-exponential constant ($m^2 \cdot s^{-1}$), E_a is the activation energy (eV or $kJ.mol^{-1}$), R is the molar universal gas constant (=8.314 J $mol^{-1}.K^{-1}$), and T is temperature (K).

The relationship between $\ln D_{Li}$ and 1/T is linear and the activation energy can be obtained from the slope of the fitting line.

Supplementary Note 4

Phase-field simulation method

A phase-field model is developed to simulate the Li dendrite growth behavior from both untreated and treated Li anodes. A phase-field variable (ξ), whose value changes continuously from 0 to 1, is used to describe the Li metal phase ($\xi = 11$) and the liquid electrolyte ($\xi = 0$). The total free energy of the system is expressed by:

$$F = \int_V [f_{ch}(\xi, C_{Li}) + f_{grad}(\nabla\xi) + f_{elec}(C_{Li}, \phi)] \, dV \quad (4)$$

where f_{ch} is the Helmholtz free energy density, f_{grad} is the gradient energy density associated with surface energy, and f_{elec} is the electrostatic energy density. C_{Li} and ϕ denote the local Li ion (Li^+) concentration and the electrical potential. Detailed expressions of these energy densities can be found in Ref. [1]. The Li dendrite growth is described by the temporal evolution of the phase-field variable ξ , by solving the following governing equation[2]

$$\frac{\partial\xi}{\partial t} = -L_\sigma(g'(\xi) - \kappa \nabla^2\xi) - L_\eta h'(\xi)\left\{exp\left[\frac{\alpha zF\eta}{RT}\right] - C_{Li}\, exp\left[-\frac{\beta zF\eta}{RT}\right]\right\} \quad (5)$$

where t is time, L_σ is interfacial mobility, and L_η is the electrodeposition reaction constant. $g'(\xi)$ is the first derivative of a double well function $g(\xi) = W\xi^2(1-\xi)^2$, in which W is the barrier height. κ is the gradient energy coefficient, $h'(\xi)$ is the first derivative of an interpolating function $h(\xi) = \xi^3(6\xi^2 - 15\xi + 10)$. α and β are symmetric factors, where $\alpha + \beta = 1$, z is the charge number of Li ion, F is the Faraday constant, η is the overpotential, R is a universal gas constant, and T is the absolute temperature.

The temporal evolution of the Li ion concentration (C_{Li}) is obtained by solving the Nernst-Planck equation,

$$\frac{\partial c_{Li}}{\partial t} = \nabla \cdot [D^{eff} \nabla c_{Li} + \mu_{Li} c_{Li}\, zF \nabla \phi] - K\frac{\partial \xi}{\partial t} \tag{6}$$

where D^{eff} is the effective Li ion diffusivity. Its value is determined by the interpolating function $h(\xi)$, i.e., $D^{eff} = D^e(h(\xi) = 1)$, $D^{eff} = D^s(h(\xi) = 0)$, $D^{eff} = D^i(0 < h(\xi) < 1)$ where D^e, D^s and D^i denote the Li-ion diffusivity in the electrode, electrolyte, and the electrode-electrolyte interface, respectively. $K\frac{\partial \xi}{\partial t}$ is the source term for Equation 6, where K is the accumulation constant.

Assuming that the total charge remains neutral in the system, the conservation of current density is expressed by the current continuity equation,

$$\nabla \cdot (\sigma^{eff} \nabla \phi) = R\frac{\partial \xi}{\partial t} \tag{7}$$

where $\sigma^{eff} = \sigma^e h(\xi) + \sigma^s(1 - h(\xi))$ is the effective electrical conductivity of the system, σ^e and σ^s are the electrical conductivities of electrode and electrolyte, respectively. $R = c_s z\eta F$ is the current constant for source term, which describes the charge entering or leaving the system due to the chemical reactions, c_s is the site density of Li metal.

Supplementary Table 1. The estimated energy for the Li migration in α -Li_3N with different paths.

Path *i*

Distance (Å)	Normalized	Energy (eV)
0.000	0.000	0.000
0.185	0.208	0.004
0.371	0.417	0.011
0.543	0.610	0.010
0.716	0.804	0.004
0.890	0.890	0.000

Path *ii*

Distance (Å)	Normalized	Energy (eV)
0.000	0.000	0.000
0.445	0.122	0.159
1.104	0.302	0.671
1.831	0.500	1.047
2.385	0.651	0.811
2.916	0.797	0.373
3.366	0.919	0.082
3.661	1.000	0.000

Path *iii*

Distance (Å)	Normalized	Energy (eV)
0.000	0.000	0.000
0.291	0.076	0.019
0.687	0.180	0.156
1.083	0.283	0.368
1.490	0.390	0.553
1.912	0.500	0.630
2.432	0.636	0.514
2.927	0.766	0.264
3.415	0.894	0.046
3.821	1.000	0.000

Path *iv*

Distance (Å)	Normalized	Energy (eV)
0.000	0.000	0.000
0.456	0.100	0.083
1.047	0.229	0.335
1.659	0.363	0.454
2.279	0.499	0.569
2.790	0.611	0.481
3.285	0.719	0.386
3.777	0.827	0.240
4.246	0.930	0.034
4.567	1.000	0.000

Path *v*

Distance (Å)	Normalized	Energy (eV)
0.000	0.000	0.000
0.160	0.027	0.028
0.773	0.129	0.516
1.522	0.254	1.339
2.260	0.378	1.590
2.993	0.500	1.769
3.726	0.622	1.590
4.464	0.746	1.339
5.213	0.871	0.516
5.826	0.973	0.028
5.986	1.000	0.000

Supplementary References

1. Ren Y, Zhou Y, Cao Y. Inhibit of lithium dendrite growth in solid composite electrolyte by phase-field modeling. *The Journal of Physical Chemistry C* **124**, 12195-12204 (2020).
